# Music recognition based on diffractive neural networks

**DOI:** 10.1016/j.isci.2026.115528

**Published:** 2026-03-30

**Authors:** Xingcen Ge, Zirui Feng, Qian Ma

**Affiliations:** 1 Communication University of China, Nanjing, China; 2 State Key Laboratory of Millimeter Wave, Southeast University, Nanjing 210096, China

**Keywords:** optical signal processing, computer science, artificial intelligence

## Abstract

Diffractive neural networks offer high-speed and energy-efficient computation for artificial intelligence hardware, yet their application to time-series signal processing remains underexplored. Here, we propose an electromagnetic diffractive neural network framework for music style and sentiment classification. Time-series audio signals are converted into log-Mel spectrograms and processed using a multilayer metasurface-based DNN. Experiments on the GTZAN dataset achieved classification accuracies of 90.3% for five genres and 96.33% for three genres. Furthermore, sentiment classification on the POP909 dataset achieved 90.56% accuracy. These results demonstrate that diffractive neural networks can effectively process time-series data and provide a promising hardware-efficient solution for intelligent signal processing in communication and sensing applications.

## Highlights

• Diffractive neural networks enable optical music classification
• Log-Mel spectrograms encode audio for electromagnetic processing
• Passive metasurfaces perform classification via wave propagation
• High accuracy achieved in genre and emotion classification

## Graphical abstract

**Figure:**
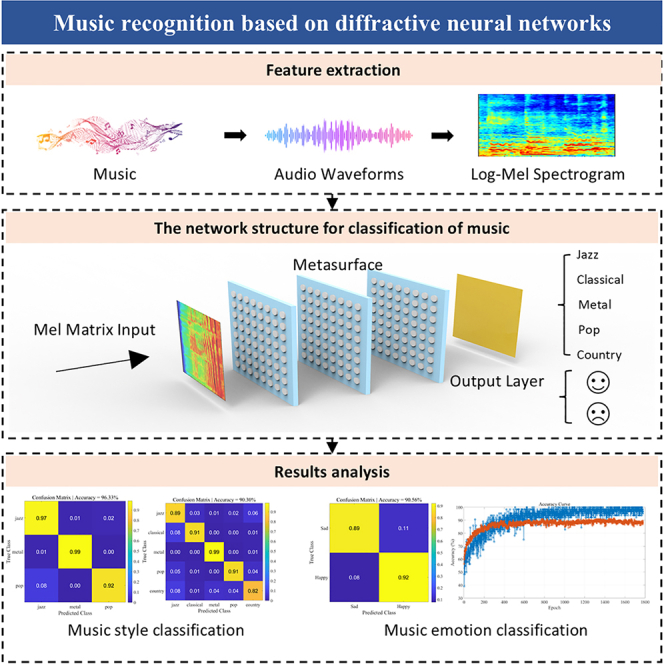

## Introduction

In recent years, the rapid advancement and widespread adoption of artificial intelligence (AI) technologies, particularly large language models, have substantially increased demand for AI computing power. Conventional electronic computing systems, constrained by transistor scaling limitations, face growing challenges in balancing power efficiency with performance gains. Addressing this problem, all-optical diffraction neural networks (DNNs)^1^^,^^2^ have emerged as a promising AI computing paradigm. Leveraging electromagnetic waves for computation, DNNs offer exceptional energy efficiency, high parallelism, and ultra-fast matrix operations.^3^^,^^4^^,^^5^^,^^6^^,^^7^ Numerous DNN implementations now demonstrate capabilities across diverse AI tasks, including image classification/recognition,^8^^,^^9^^,^^10^ target sensing,^11^ and electromagnetic signal processing.^12^^,^^13^^,^^14^^,^^15^ As an artificially engineered electromagnetic medium, metamaterials provide a robust platform for realizing such networks. Over two decades of development, metamaterials have achieved precise control over electromagnetic wave amplitude,^16^ phase,^17^^,^^18^ polarization,^19^ and frequency.^20^ Recent advances confirm that metamaterial-based DNNs can execute complex AI tasks in the microwave regime, such as spatial electromagnetic signal encoding/decoding,^12^^,^^13^^,^^14^ image classification,^21^ and adaptive field control.^12^

Although DNN-based intelligent computing and recognition have developed many functions, the classification of music styles and emotions based on DNN has not been deeply studied and verified. As a typical time-domain sequence information, music has been studied to a certain extent in the field of machine learning based on traditional computers. Liang et al., based on a hierarchical learning framework, can achieve melody completion, accompaniment selection and genre classification by modeling note sequences and integrating pitch, rhythm and intensity information.^22^ George Tzanetakis proposed and established the GTZAN dataset containing 10 genres, and achieved style classification by extracting three features: timbre, rhythm and pitch.^23^ Another method is to convert the audio signal into feature images of different forms and use convolutional neural networks to classify the audio style.^24^^,^^25^^,^^26^ In addition, music can be sliced into different forms, and segment features can be extracted through semantic models for classification.^27^^,^^28^ As a new AI computing hardware architecture, DNN has also been shown to have relatively good performance in image recognition, particularly due to its excellent energy efficiency and high computing power.^2^ Previously, diffractive neural network architectures were mostly based on spatial diffraction,^12^^,^^29^ with input information, such as images, being fed into the network in the form of one- or two-dimensional matrices for processing.^12^^,^^13^^,^^14^^,^^30^ However, music signals are time-varying sequences, and how to efficiently process and recognize them in DNNs is a key challenge.

We proposed a DNN-based method for music style and emotional detail classification, demonstrating how this physical hardware architecture can process time sequence signals. We performed slice classification on samples from the music style dataset GTZAN. Using log-Mel feature extraction, we transformed the music sequence signal from the time domain to the frequency domain, thereby converting the time series signal into an image-like data signal. We designed electromagnetic DNNs of different scales and layers to match the requirements of different classification tasks. Our classification accuracy for five music styles was 90.3%, and for three styles was 96.33%. Furthermore, based on this method, we also verified two music emotion classifications and achieved an accuracy of 90.56%. More importantly, we validate a hardware computing paradigm for time-series signals that promises ultra-low latency and high energy efficiency compared to digital electronic processors. This demonstration paves the way for applying diffractive computing to a broader class of temporal signals, such as those in wireless communications and radar systems.

## Results

**Figure 1:**
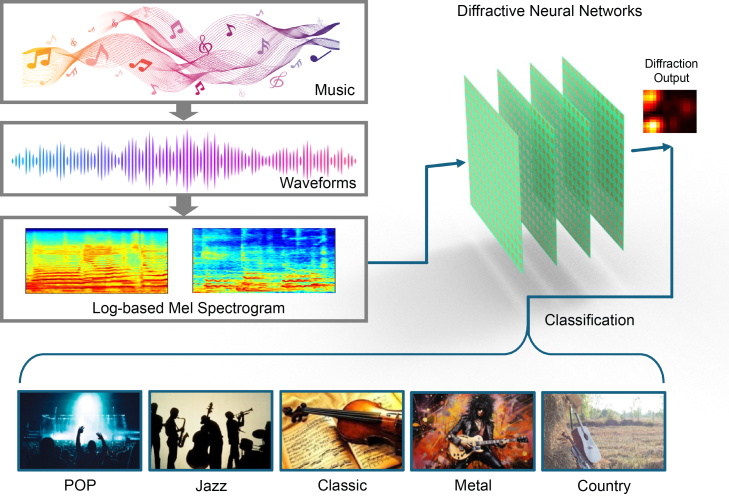
Electromagnetic diffractive neural network framework for music classification Schematic illustration of the electromagnetic diffractive neural network for music classification. Time-domain audio signals are first converted into log-Mel spectrogram matrices, which encode time-frequency features of the music signals. These spectrograms are used as spatial input patterns and injected into the diffractive neural network. The network consists of multiple cascaded metasurface layers that modulate the phase of incident electromagnetic waves during propagation. The output intensity distribution at the detection plane corresponds to classification probabilities across predefined output regions. This configuration enables passive optical computation for music classification. The example shown illustrates the classification of five music genres.

First, we introduce the concepts and principles of music recognition based on DNNs, as shown in Figure 1. DNNs are inherently adept at processing spatial diffraction information, which in optics can typically be viewed as a planar diffraction field (i.e., a planar image). The advantage of DNNs lies in the ability to simultaneously input and process these planar light field signals (the computation is complete once the diffraction process completes). Music, on the other hand, is a time-series signal (i.e., a waveform), and its single-point input signal varies over time. Therefore, the key lies in converting these waveform signals into a planar light field matrix and enabling the neural network to extract useful information. As shown in Figure 1, we first convert the music signal into a planar image based on log-Mel features. This image uses nonlinear spectral sampling to mimic the way the human ear captures sound characteristics. This image can then be used as a planar light field input via a programmable metasurface or spatial light modulator. This input information passes through a four-layer DNN, where it converges at the output end to a specific energy level, which serves as the classification result. For example, we have implemented classification for five musical styles (Jazz, Classical, Metal, Pop, and Country). Five convergence points can be set at the network input end, and the point with the highest converged energy is used as the classification result.

**Figure 2:**
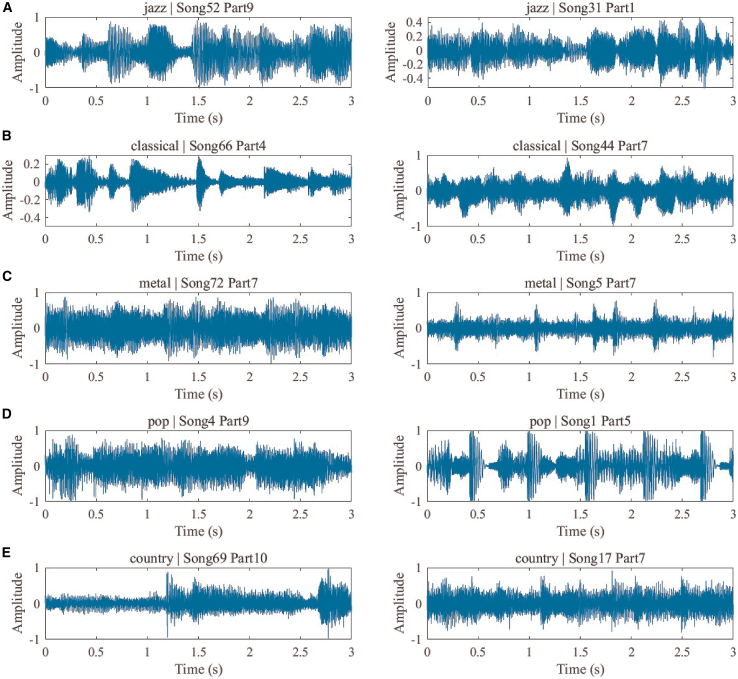
Representative waveform signals for music genre classification Time-domain waveform signals from the GTZAN dataset are used for music genre classification. Five music genres are shown: Jazz (A), Classical (B), Metal (C), Pop (D), and Country (E). Two representative audio samples are displayed for each genre. These waveforms illustrate differences in amplitude variation, temporal structure, and signal characteristics among different music genres, which form the basis for feature extraction and classification.

**Figure 3:**
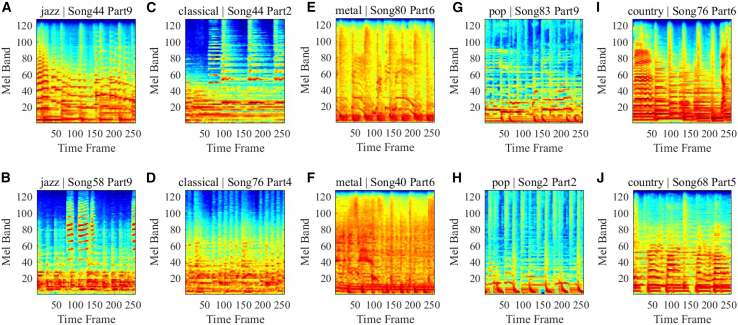
Log-Mel spectrogram representations used as input to the diffractive neural network Log-Mel spectrogram matrices derived from the waveform signals shown in Figure 2. Five music genres are presented: Jazz (A and B), Classical (C and D), Metal (E and F), Pop (G and H), and Country (I and J). Each spectrogram represents the time-frequency distribution of signal energy. These spectrograms serve as spatial input patterns to the diffractive neural network, enabling electromagnetic wave-based feature processing and classification.

Figure 2 shows five typical musical styles, which serve as targets for our DNN classification and recognition. Music has evolved into a vast array of styles. Early music before the Renaissance was primarily vocal, until the Baroque period, with its structures and concertos, produced more ornate and contrasting melodies (such as Bach’s “The Well-Tempered Clavier”). The Classical period, exemplified by Mozart and Beethoven, emphasized balance and formal beauty. Modern Romanticism (19th century) emphasized personal emotion and national characteristics, as exemplified by Schubert, Chopin, and Wagner. In modern times, musical styles have become increasingly fragmented and diverse. From Debussy’s Impressionism to more modern jazz, rock, and electronic pop, they incorporate a wider range of technologies and cultures. We have selected five representative musical styles, as shown in Figure 2: Jazz, Classical, Metal, Pop, and Country. Figures 2A–2E show typical music clips from these five styles, showing the different musical styles in the form of audio signal waveforms. To further illustrate their spectral characteristics, Figure 3 lists the corresponding Mel matrices for the clips in Figure 2. We can see from its time-frequency domain characteristics that different music styles form distinct spectral responses through the combination of rhythm, melody, and instruments.

**Figure 4:**
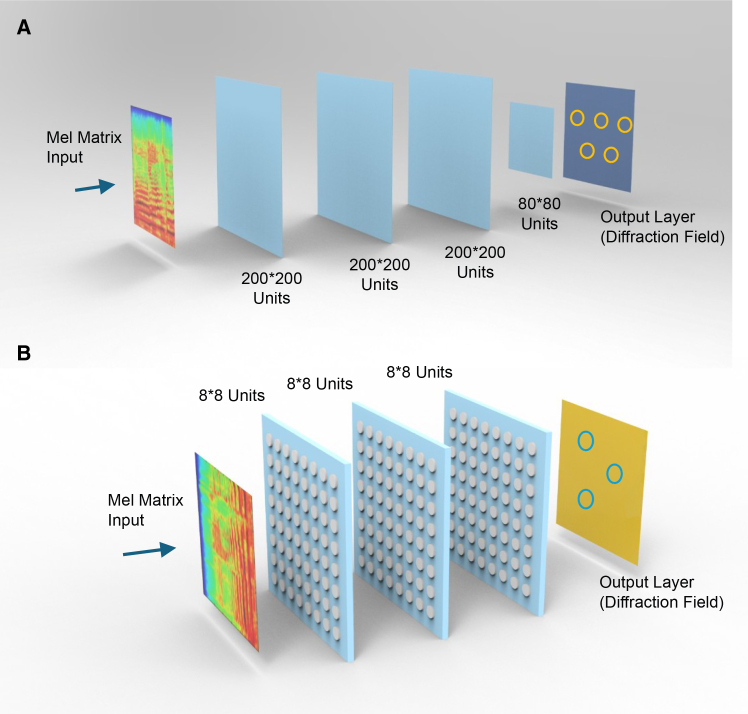
Diffractive neural network architectures for music genre classification Structure of the diffractive neural network used for classification tasks. (A) Network configuration for five-genre classification, where the output plane is divided into five spatial detection regions corresponding to Jazz, Classical, Metal, Pop, and Country. (B) Network configuration for three-genre classification, where the output plane contains three detection regions corresponding to Jazz, Metal, and Pop. Each network consists of multiple metasurface layers with trainable phase modulation elements. The classification result is determined by the output region with the highest optical intensity.

To achieve the classification of the aforementioned music styles, we designed a DNN structure, as shown in Figure 4. The input information is first preprocessed from the original audio clip into a 128×250 Mel matrix. This process refers to the information preprocessing performed using a Mel eigenfilter.^23^ Subsequently, the intensity information of this matrix is fed into a transmissive metasurface with programmable amplitude. The metasurface recognition network consists of 3 or 4 layers of phase control metasurface, with the network center frequency set to 24 GHz. Phase-shift neurons utilize multi-layer metal patch-type transmissive metasurface units,^21^ which can achieve 360-degree phase shifts. The output layer (i.e., the output diffraction field) is configured with 3–5 convergence points to achieve musical style classification. The matrix intensity values correspond to the unit amplitude values of the metasurface in the input layer, enabling the injection of electromagnetic information. In the GTZAN dataset, the five musical styles we selected contain 500 music tracks, each of which can be divided into 10 30-s segments (each segment corresponds to a Mel matrix), totaling 5,000 data points. After randomly sorting the data, 80% of the samples were selected as the training set and 20% of the samples were selected as the test set. In the test for the five-style classification of music, the diffraction network had four intermediate layers, as shown in Figure 4A. The first three layers consisted of 200∗200 metasurface units, and the fourth layer consisted of 80∗80 units. The output layer (i.e., the output diffraction field) had five convergence points to achieve music style classification. In the test for the three-style classification of music, the diffraction network had three intermediate layers, as shown in Figure 4B. All three layers consisted of 8∗8 metasurface units, and the output layer (i.e., the output diffraction field) had three convergence points to achieve classification.

**Figure 5:**
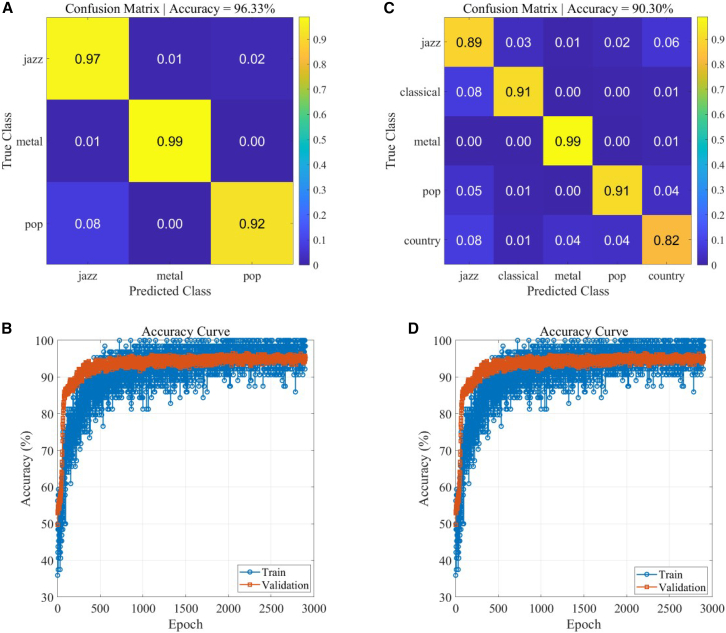
Classification performance of the diffractive neural network for music genres Performance evaluation results for music genre classification using the diffractive neural network. (A) Confusion matrix shows classification accuracy for three music genres: Jazz, Metal, and Pop. (B) Training progress curve shows classification accuracy versus training iterations for the three-genre classification task. (C) Confusion matrix shows classification accuracy for five music genres: Jazz, Classical, Metal, Pop, and Country. (D) Training progress curve shows classification accuracy versus training iterations for the five-genre classification task. These results demonstrate that the diffractive neural network successfully learns discriminative features and achieves high classification accuracy across multiple genre classification scenarios.

Figure 5 shows the results of music style classification. Two sets of experiments are presented here. First, the network classification of three music styles, Jazz, Metal, and Pop, achieved a recognition accuracy of 96.33%. The confusion matrix is shown in Figure 5A. The diffraction network was configured with three layers, each with 64 neurons (8∗8). Training was performed with 3000 iterations, and the convergence curve of the training process is shown in Figure 5B. To further verify the network’s performance, Figure 5C shows the classification results of five music styles, achieving an accuracy of 90.3%. The diffraction network was configured with four layers, with neurons of 200,^2^ 200,^2^ 200,^2^ and 80^2^, respectively. The convergence curve of the training process is shown in Figure 5D. As can be seen from the figure, increasing the number of music style categories significantly increases the classification difficulty, resulting in a decrease in accuracy. However, with appropriate network parameter settings, accuracy can still exceed 90%.

**Figure 6:**
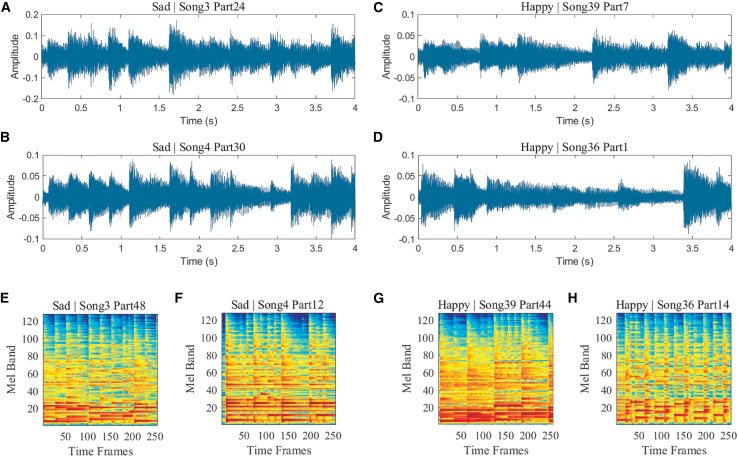
Representative waveform and spectrogram data for music emotion classification Example audio signals and corresponding spectrogram representations used for emotion classification based on the POP909 dataset. (A and B) Time-domain waveforms of two audio clips labeled as sad emotion. (C and D) Time-domain waveforms of two audio clips labeled as happy emotion. (E and F) Log-Mel spectrograms corresponding to the sad audio clips. (G and H) Log-Mel spectrograms corresponding to the happy audio clips. These spectrograms serve as input patterns to the diffractive neural network for emotion classification.

To further validate the network’s performance, we also performed music emotion classification. Based on the POP909 dataset,^31^ we set two basic emotion labels (happy and sad). This dataset contains 909 songs. Considering the significant disparity in the number of songs with happy and sad labels, we selected 40 songs (half happy and half sad). Each song was sliced into 4-s segments and preprocessed into a log-Mel feature matrix. The slice waveforms and Mel matrix are shown in Figure 6. Happy and sad styles exhibit different spectrum-time relationships. Figure 6 shows the time-domain waveforms and Mel feature matrices of some of these segments.

**Figure 7:**
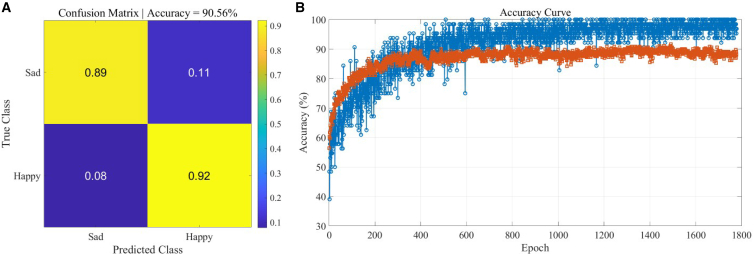
Classification performance for music emotion recognition Results of binary classification between happy and sad emotional categories. (A) Confusion matrix shows classification accuracy for the emotion classification task. (B) Training progress curve shows classification accuracy as a function of training iterations. The results demonstrate that the diffractive neural network can successfully distinguish emotional characteristics of music signals based on spectrogram input features.

The network structure is the same as that shown in Figure 4B. Here, the diffraction network is set to three layers, with 64 neurons (8∗8) in each layer. The dataset contains 1800 samples, 80% of which are randomly selected as the training set, and 20% as the test set. The network’s recognition accuracy is 90.56%, and its confusion matrix is shown in Figure 7A. The number of training iterations is 1800, and the convergence curve of the training process is shown in Figure 7B.

### Comparison with existing methods

To comprehensively evaluate the performance of our proposed DNNs, we conducted a comparative analysis against several established software-based algorithms on the same GTZAN (for genre) and POP909 (for emotion) datasets, using identical log-Mel spectrogram features. We compare three key metrics: classification accuracy, inference latency, and energy consumption per inference. The software-based models were benchmarked on a standard high-performance computing platform (Intel Ultra 9 desktop processor with NVIDIA RTX 5080 GPU), while the DNN metrics are derived from its physical operating principles.

As summarized in Table 1, our DNN achieves competitive classification accuracy against representative software methods, including SVM, CNN, LSTM, and Transformer models, under consistent experimental settings. The accuracy values for the baseline methods are drawn from or are consistent with prior studies on similarly processed data [28–31]. The distinctive advantage of our hardware-based approach, however, becomes unequivocally clear when examining computational efficiency. Table 2 details the measured inference time and estimated energy consumption per sample for each method. The DNN executes inference at the speed of light through passive diffraction, resulting in a latency of about 10 μs, orders of magnitude faster than the millisecond-range latencies of GPU-accelerated software models. More critically, the DNN’s energy consumption per inference is estimated at about 0.05 μJ, which is a factor of 10^4^ to 10^7^ lower than the joule-level consumption of its digital counterparts. This extraordinary efficiency stems from the DNN’s elimination of power-intensive digital logic and memory access bottlenecks, performing computation directly in the analog domain. It should be noted that the inference time for software models is measured as end-to-end latency per sample, including data transfer and GPU kernel execution. Power consumption is estimated based on average GPU/CPU power draw during inference and the per-sample processing time. The physical computation of DNN is nearly instantaneous, with system latency and power dominated by electronic interfacing.

**Table 1:** Performance comparison of different methods on music classification tasks in terms of classification accuracy

Method	Type	Model/Kernel	Accuracy (5 Genres)	Accuracy (3 Genres)	Accuracy (Emotion)	Reference/Notes
**Proposed DNN**	**Hardware (Optical)**	4-layer Metasurface- ANN	**90.3%**	**96.33%**	**90.56%**	This work
Support Vector Machine (SVM)	traditional ML	RBF Kernel	∼85–87%	∼91–93%	∼85–88%	Tzanetakis, and Cook^23^
Convolutional Neural Network (CNN)	deep learning	ResNet-style CNN	∼91–93%	∼96–97.5%	∼91–93%	Hershey et al.^32^; Choi et al.^33^
Long Short-Term Memory (LSTM)	deep learning	2-layer LSTM	∼87–90%	∼93–95%	∼88–91%	Choi et al^34^
Transformer	deep learning	4-layer Encoder	92.5%∗	97.2%∗	93.0%∗	*Implemented for comparison*

Bold entries indicate the corresponding data of the proposed architecture in this work.

**Table 2:** Comparison of inference speed and energy consumption per inference for different methods

Method	Inference Time (per sample)	Energy per Inference	Notes
**Proposed DNN**	**∼10 μs**	**∼0.05 μJ**	latency dominated by electronic readout; energy from peripheral components (5 mW average power).
SVM (RBF Kernel)	∼1–3 ms	∼0.25–0.75 J	CPU-bound; energy estimated using a 250 W system power.
CNN (ResNet-18)	∼2–5 ms	∼1.5–3.0 J	GPU-accelerated; energy estimated using a 250 W system power.
LSTM (2-layer)	∼6–12 ms	∼1.25–2.5 J	sequential processing; energy estimated using a 250 W system power.
Transformer (4-layer)	∼8–15 ms	∼2–3.75 J	computationally intensive; energy estimated using a 250 W system power.

Bold entries indicate the corresponding data of the proposed architecture in this work.

Therefore, while Table 1 confirms that our DNN delivers accuracy on par with state-of-the-art software models, Table 2 reveals its transformative potential as a high-throughput, ultra-low-power hardware accelerator. This combination of competitive accuracy with radical efficiency gains underscores the promise of diffractive computing for real-time, energy-constrained processing of time-series signals such as audio, with direct implications for future communication and embedded sensing systems.

## Discussion

This work demonstrates the feasibility of using electromagnetic diffractive neural networks for time-series signal classification tasks. By transforming audio signals into log-Mel spectrogram representations, the proposed system leverages spatial optical computing for feature processing. Compared with traditional electronic neural networks, the diffractive neural network offers advantages in energy efficiency and inference speed due to its passive propagation mechanism. These characteristics make diffractive neural networks promising candidates for low-power and high-speed AI hardware systems, particularly in applications requiring real-time signal analysis.

In this study, a metasurface-based electromagnetic diffractive neural network was designed to classify musical genres and emotional labels. Music signals, as representative time-series data, were converted into image-like matrix representations through frequency feature extraction. These log-Mel spectrogram matrices were processed by multilayer transmissive metasurfaces, enabling electromagnetic wave-based computation and classification. The system achieved recognition accuracies of 90.3% and 96.33% for five-genre and three-genre classification tasks, respectively. In addition, the binary classification of musical emotions achieved an accuracy above 90%, demonstrating the capability of the diffractive neural network framework for multiple time-series signal processing tasks.

However, the system performance depends on feature encoding quality and metasurface design precision. The classification accuracy is influenced by the representation capability of the log-Mel spectrogram and the optimization of the diffractive layers. Future work may focus on improving classification accuracy through optimized network topology, increased diffractive layer depth, and enhanced training strategies. Furthermore, hybrid optical-electronic architectures may provide improved flexibility and scalability while maintaining computational efficiency. Extending this framework to real-time hardware implementations and broader signal processing tasks such as radar signal analysis, wireless communication signal classification, and spectrum sensing represents a promising direction for future research.

### Limitations of the study

Despite the promising results demonstrated in this work, several limitations should be considered. First, the proposed framework relies on converting time-series signals into log-Mel spectrogram representations, which may introduce information loss and limit the ability of the diffractive neural network to capture certain temporal dependencies present in raw signals. Second, the performance of the system is closely related to the precision of metasurface design and fabrication. In practical hardware implementations, fabrication errors, material losses, and alignment inaccuracies may degrade the system performance compared to simulation results.

In addition, the current study focuses on relatively small-scale classification tasks using standard benchmark datasets. The scalability of the diffractive neural network to larger datasets, more complex classification tasks, and higher-resolution input representations remains to be further investigated. Moreover, the present work primarily demonstrates a computational framework rather than a fully integrated real-time hardware system. Future work is needed to validate the proposed approach in experimental electromagnetic or optical hardware platforms and evaluate its robustness under practical operating conditions. Finally, compared with conventional electronic neural networks, diffractive neural networks may have limitations in flexibility, particularly in tasks requiring frequent model updates or adaptive learning, since the physical reconfiguration of metasurfaces can be more challenging than updating software-based models.

## Resource availability

### Lead contact

Further information and requests for resources should be directed to and will be fulfilled by the Lead Contact, Qian Ma (maqian@seu.edu.cn).

### Materials availability

This study did not generate new unique physical materials. The diffractive neural network models were designed and simulated using computational electromagnetic and deep learning frameworks.

### Data and code availability

• Data: All data used in this study are publicly available. The GTZAN dataset is available at http://marsyas.info/downloads/datasets.html, and the POP909 dataset is available at https://github.com/music-x-lab/POP909-Dataset.
• Code: This paper reports original custom code. The code is available from the lead contact upon request.
• Additional information: Any additional information required to reanalyze the data reported in this paper is available from the lead contact upon request.

## Acknowledgments

The work was supported by the 10.13039/501100001809National Natural Science Foundation of China (62301147, and 62288101), the 10.13039/501100004608Natural Science Foundation of Jiangsu Province (BK20230822), Young Elite Scientists Sponsorship Program by CAST (2022QNRC001), the 10.13039/501100011421State Key Laboratory of Millimeter Waves of Southeast University, China (K201924), the 10.13039/501100012226Fundamental Research Funds for the Central Universities (2242023K5002), and the 10.13039/501100002858China Postdoctoral Science Foundation (2021M700761, 2022T150112).

## Author contributions

X.G. designed the study and conducted experiments. Z.F. contributed to model design and data processing. Q.M. supervised the research and wrote the manuscript. All co-authors have read and approved the final version of the manuscript.

## Declaration of interests

The authors declare no competing interests.

## STAR★Methods

### Key resources table

**Table:** 

REAGENT or RESOURCE	SOURCE	IDENTIFIER
**Deposited data**		

GTZAN dataset	Marsyas	http://marsyas.info/downloads/datasets.html
POP909 dataset	Music X Lab	https://github.com/music-x-lab/POP909-Dataset

**Software and algorithms**		

MATLAB	MathWorks	https://www.mathworks.com
Custom diffractive neural network code	This paper	Available from Lead Contact

### Method details

#### Audio signal preprocessing and feature extraction

The input audio signals were first converted into log-Mel spectrogram representations to enable spatial encoding for diffractive neural network processing. Each audio signal was segmented into fixed-duration samples and transformed using short-time Fourier transform (STFT). The Mel filter bank was applied to the power spectrum to generate Mel-frequency representations, which were then converted into logarithmic scale to obtain log-Mel spectrograms.

The resulting spectrograms were resized to match the input resolution required by the diffractive neural network. These spectrograms served as spatially encoded input signals representing time-frequency features of the original audio data.

#### Diffractive neural network architecture

The diffractive neural network consists of multiple cascaded metasurface layers designed to manipulate electromagnetic wave propagation. Each metasurface layer contains a two-dimensional array of trainable phase modulation units. These units modulate the phase of incident electromagnetic waves, enabling the network to perform computation through wave propagation and interference.

The propagation between layers was modeled using scalar diffraction theory. The output plane captures the resulting intensity distribution, which corresponds to classification probabilities across predefined output regions.

#### Network training and optimization

The diffractive neural network parameters were optimized using supervised learning. The phase modulation values of metasurface units were treated as trainable parameters. The network was trained using backpropagation and gradient-based optimization to minimize cross-entropy loss between predicted and ground truth labels.

The training process was conducted using standard deep learning frameworks with custom electromagnetic propagation layers implemented to simulate physical wave propagation. The trained network parameters were then used to evaluate classification performance on test datasets.

#### Dataset description and experimental setup

For genre classification, the GTZAN dataset containing 10 music genres was used. Subsets of five and three genres were selected to evaluate classification performance under different complexity levels.

For sentiment classification, the POP909 dataset was used. Music clips were categorized into sentiment classes based on emotional characteristics.

The datasets were divided into training and testing sets. Log-Mel spectrograms were generated for each sample and used as input to the diffractive neural network.

### Quantification and statistical analysis

Classification performance was evaluated using accuracy as the primary metric. Accuracy was defined as the ratio of correctly classified samples to the total number of samples in the test set.

Training and testing were conducted using standard dataset splits. The reported classification accuracies represent the performance of the trained diffractive neural network on unseen test data.

No additional statistical hypothesis testing was performed, as the objective of this study was to demonstrate feasibility and classification performance of the diffractive neural network framework.

### Additional resources

This study did not generate additional external resources.
